# The effects of supplemental melatonin administration on the healing of bone defects in streptozotocin-induced diabetic rats

**DOI:** 10.1590/1678-775720150570

**Published:** 2016

**Authors:** Senem YILDIRIMTURK, Sule BATU, Canan ALATLI, Vakur OLGAC, Deniz FIRAT, Yigit SIRIN

**Affiliations:** 1 - Istanbul University, Faculty of Dentistry, Department of Oral and Maxillofacial Surgery, Istanbul, Turkey.; 2 - Istanbul University, Faculty of Dentistry, Department of Biochemistry, Istanbul, Turkey.; 3 - Istanbul University, Institute of Oncology, Department of Pathology, Istanbul, Turkey.

**Keywords:** Bone, Free radicals, Diabetes mellitus, Melatonin, Rats

## Abstract

Diabetes mellitus (DM) causes an increased production of free radicals that can impair bone healing. Melatonin is a hormone secreted mainly by the pineal gland, which participates in the neutralization process of free radicals.

**Objective:**

The aim of this study was to investigate histologic and biochemical effects of supplemental melatonin administration on bone healing and antioxidant defense mechanism in diabetic rats.

**Material and Methods:**

Eighty-six Sprague-Dawley male rats were used in this study. Diabetes mellitus was induced by intraperitoneal (i.p.) administration of 65 mg/kg streptozotocin (STZ). Surgical bone defects were prepared in the tibia of each animal. Diabetic animals and those in control groups were treated either with daily melatonin (250 μg/animal/day/i.p.) diluted in ethanol, only ethanol, or sterile saline solution. Rats were humanely killed at the 10^th^ and 30^th^ postoperative days. Plasma levels of Advanced Oxidation Protein Products (AOPP), Malondialdehyde (MDA), and Superoxide Dismutase (SOD) were measured. The number of osteoblasts, blood vessels and the area of new mineralized tissue formation were calculated in histologic sections.

**Results:**

At the 10^th^ day, DM+MEL (rats receiving both STZ and melatonin) group had significantly higher number of osteoblasts and blood vessels as well as larger new mineralized tissue surfaces (p<0.05 for each) when compared with DM group. At the 30^th^ day, DM group treated with melatonin had significantly lower levels of AOPP and MDA than those of DM group (p<0.05).

**Conclusion:**

Melatonin administration in STZ induced diabetic rats reduced oxidative stress related biomarkers and showed beneficial effects on bone healing at short term.

## INTRODUCTION

Diabetes mellitus (DM) is a group of metabolic diseases characterized by hyperglycemia resulting from defects in insulin secretion, insulin action, or both^5^. It is caused by either autoimmune destruction of insulin-producing cells (Type I) or resistance of the body to insulin (Type II). Diabetes mellitus causes long term chronic complications, such as increased fracture risk, poor osseous healing characteristics and impaired bone regeneration potential, by modulating oxidative stress in various systems^25^.

Oxidative stress generates mainly from glucose autoxidation, which leads to free radical and reactive oxygen species (ROS) formations. These formations are routinely produced as products of normal cellular metabolism in aerobic organisms. Since free radicals have beneficial and harmful effects, it is of utmost importance to maintain the balance between the reactive species and radical scavenging mechanisms known as antioxidants. Oxidative stress derives from overproduction of ROS and the inadequacy of enzymatic and non-enzymatic antioxidant defense mechanisms that lead to an imbalance in the equilibration of cellular oxidation/reduction. Diabetes mellitus leads to hyperglycemia, enhanced and prolonged inflammation as well as generation of ROS, which in turn may adversely affect bone healing through enhanced expression of pro-inflammatory cytokines that reduce osteoblast differentiation, osteoblast activity and increase osteoblast apoptosis. Reactive oxygen species induce a broad range of responses including proliferation, growth, differentiation and cell death by activating numerous signaling pathways^14^. Excessive production of ROS induced by hyperglycemia causes deleterious effects on lipids and proteins by altering their structures and functions^25^. These molecules participate in the conversion of non-radical lipid molecules to radicals, which leads to a chain reaction called lipid peroxidation. Lipid peroxidation products could impair the flexibility, permeability, integrity, and fluidity of the cell membrane. A well-established marker of lipid peroxidation, the Malondialdehyde (MDA), is a low molecular weight end-product of this process that can be measured by spectrophotometry^6^. Advanced oxidation protein products (AOPPs) are dityrosine-containing, cross-linking protein products described as novel markers of oxidant-mediated protein damage. Advanced oxidation protein product is formed by the action of chlorinated oxidants produced by myeloperoxidase in activated neutrophils. This product is considered a reliable marker to evaluate the degree of protein oxidation, and is accumulated in biological systems. Increased plasma levels of AOPP may be observed in long-term diabetic complications that cause structural and functional damage in biological membranes and endothelium^23^. To counter harmful effects of excess free radicals, organisms develop antioxidant defense mechanisms that consist of enzymatic and non-enzymatic reactions. Superoxide dismutase (SOD) enzyme activity enhances the spontaneous dismutation of superoxide anion to hydrogen peroxide and oxygen molecules, which decompose to oxygen and water. During oxidative burst, the activity and level of SOD increase in tissues. Current research is focused on reducing diabetes induced ROS damage using various free radical-scavenging enzymatic or non-enzymatic antioxidants^8^.

The radical scavenger antioxidant melatonin (N-acetyl-5-methoxytryptamine) is a hormone secreted mainly by the pineal gland, which has the ability to stimulate antioxidant enzymes that neutralize free radicals and ROS. Melatonin also contributes to the maintenance of bone health by promoting osteoblast differentiation and limiting osteoclastic activity. Bone healing process consists of inflammatory, proliferative, and remodeling phases. The production of free radicals causes cell damage and disruption of bone healing process due to the chain reactions of protein and lipid peroxidation. Melatonin participates in the physiological functions of bone cells, promotes angiogenesis and, through its free radical scavenging properties, it may also serve as a preventive agent against radical-induced hard tissue damages^12^.

Impaired wound healing in Type I DM warranted further research on supplemental substances that may provide beneficial effects in the preservation of bone structures. As far as we know, there is a limited number of studies that focus on the potential effects of melatonin on the healing of hard tissue wounds and biochemical markers of free-radical mediated damage in Type I DM. Therefore, the aim of the this study was to investigate the effects of supplemental melatonin administration on the histologic variables of bone healing process, oxidative stress and biomarkers of antioxidant defense mechanism in STZ-induced diabetic rats.

## MATERIAL AND METHODS

### Experimental animals and study groups

All procedures were reviewed and approved by the Institutional Animal Care and Use Committee of the Istanbul University, Institute for Experimental Medical Research (Project No. 2012/153). Experiments were carried out on 10-12 week-old adult male Sprague-Dawley rats (N=86) weighing approximately 300±20 g, obtained from the Institute for Experimental Medical Research. All animals were housed in metallic cages in a temperature (22±1°C) and humidity (40-60%) of a controlled room with a 12 hours dark/light cycle (lights on at 08:00). Rats were given *ad libitum* access to commercial standard chow and tap water. The body weights were daily recorded throughout the experiments. After one week of acclimatization, animals were randomly allocated to six main groups concerning the substances that will be administered alone or in combination. Since two sacrification time points (10^th^ and 30^th^ postoperative days) were determined before the experiments, the main groups were further divided into two subgroups. Therefore, there were 12 experimental groups involved in this study. Those which had only STZ injection were described as DM group (n=15), whereas rats which received both STZ and melatonin were identified as DM+MEL group (n=15). Animals injected with both STZ and ethanol were grouped under DM+ETN entity (n=14). Non-diabetic animals, which will be injected with same doses of either melatonin (MEL group, n=14), ethanol (ETN group, n=14), or sterile saline solution (CONT group, n=14), were identified as diabetic animals. See Table 1 and 2 for number of animals in each subgroup.

**Table 1:** Measurement of biochemical variables in study groups. Data were expressed as mean±standard deviation except for SOD group, in which the median values have been presented between parentheses. (AOPP: Advanced Oxidation Protein Products; MDA: Malondialdehyde; SOD: Superoxide Dismutase; DM: Diabetes Mellitus; MEL: Melatonin; ETN: Ethanol; CONT: Control)

Day	n	Groups	AOPP (μmol/L)	MDA (μmol/L)	SOD (U/ml)
10	8	DM	88.26±17.87^A,B,C^	2.41±0.24^A,B,C^	54.59±16.6 (48.3)^A^
10	8	DM+MEL	52.47±25.93	1.99±0.56	48.13±5.14 (46.7)
10	7	DM+ETN	93.52±36.71	2.3±0.28^D,E,F^	38.88±1.99 (38.7)^A^
10	7	ETN	50.56±15.02^A^	1.2±0.38^A,D^	41.33±8.62 (31.7)
10	7	MEL	49.56±15.02^B^	1.62±0.26^B,E^	43.68±7.38 (45.1)
10	7	CONT	41.73±20.64^C^	1.59±0.39^C,F^	46.18±21.6 (35)
30	7	DM	104.42±17.7^A,B,C,D^	2.55±0.08^A,B,C,D^	35.87±6.35(36.7)^A,B^
30	7	DM+MEL	29.42±20.83^A,E^	1.58±0.37^A,E^	37.57±4.27 (37.7)^C,D^
30	7	DM+ETN	98.16±34.96^E^	2.29±0.25^E,F,G^	35.75±3.36 (35.3)^E,F^
30	7	ETN	42.45±21.57^B^	1.45±0.18^B,H^	42.15±7.03(36)^G,H^
30	7	MEL	40.09±10.85^C^	1.23±0.37^C,F,H^	46.07±4.02 (45)^A,C,E,G^
30	7	CONT	42.09±10.84^D^	1.43±0.37^D,G^	45.4±2.32 (46.2)^B,D,F,H^

Upper-case letters in the same column indicate significant difference between two groups at the same time points.

### STZ preparation and administration

A dose of STZ was individually calculated for each animal. STZ powder (Streptozotocin U-9889, Santa Cruz Biotechnology Inc, Dallas, TX, USA) was transferred into microfuge tubes and maintained at -20°C. The streptozotocin was kept in unbroken cold chain and freshly dissolved in citrate buffer before administration. Animals were fasted overnight for 12 hours with free water access before STZ injections. Type I DM was induced by single intraperitoneal injection of 65 mg/kg STZ dissolved in citrate buffer (0.1 M; pH 4.5) in a volume of 0.5 mL.

### Fasting blood glucose measurement and urine analysis

Three days after diabetes induction, blood samples obtained from tail veins were analyzed for fasting blood glucose using a glucometer (Major II Blood Glucose Monitoring System, Major Biosystems Corp, New Taipei City, Taiwan). Animals were considered Type I DM if blood glucose level exceeds 200 mg/dl^1^ ^,^ ^30^. During the experiments, urine glucose measurements were weekly evaluated by using urine test strips (Urine Reagent Strip-10 URS-10, Teco Diagnostics, Anaheim, CA, USA).

### Surgical procedures

**Figure 1:**
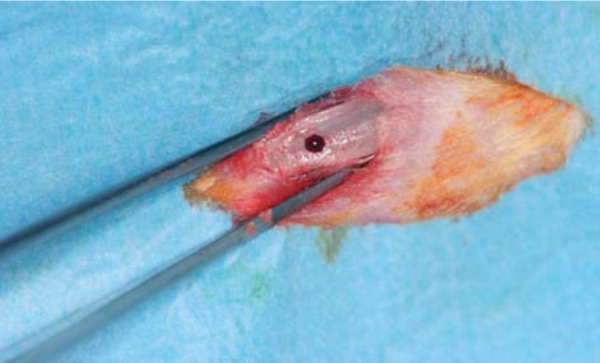
Macroscopic view of the bone defect in the right tibia

Four weeks after the induction of DM, rats were anesthetized using i.p. injection of 5 mg/kg of Xylazin hydrochloride (Rompun^®^, Bayer Turk Kimya San. Ltd. Sti. Istanbul, Turkey) and 60 mg/kg of Ketamin HCl (Ketanest^®^, Parke Davis, Berlin, Germany). Medial surfaces of right tibiae were shaved and disinfected. A two centimeter longitudinal incision was performed along the frontal aspect and bone was exposed by blunt dissection. A single, non-critical corticocancellous bone defect of 2.1 mm, both in diameter and in depth, was prepared in the right tibiae of each animal under copious amount of sterile saline irrigation using a dental bur (HM 71 021, Meisinger, Hager&Meisinger GmbH, Neuss, Germany) attached to a surgical physio dispenser (X Cube V2.0, Saeshin Precision Co, Daegu, Korea) (Figure. 1). The surgical wounds were closed with 3.0 surgical silk sutures (Dogsan Tibbi Malzeme San. A.S., İstanbul, Turkey). All injections started immediately after the surgery.

### Melatonin and ethanol administration

Melatonin (Santa Cruz Biotechnology, Dallas, TX, USA) was first dissolved in absolute ethanol (Tekkim Kimya San. Ltd. Sti. Bursa, Turkey), in physiological saline solution and then maintained at -20°C. Rats were daily injected with melatonin (250 µg/animal/day/i.p.) diluted in 1% ethanol-physiological saline solution, in a volume of 0.5 mL, between 19:00 and 20:00 h. The same dose of ethanol without additional melatonin was injected to animals in ETN group.

### Histological preparation and analysis

The rats were humanely euthanized and tibiae were excised. Samples were fixated in 10% buffered formaldehyde solution. About 8 to 12 hours after fixation, soft tissues were cleaned and specimens were decalcified in formic acid sodium nitrate solution. The regions with bone defects were sectioned and embedded in paraffin. Mid-sagittal serial sections of 5-7 μm thick were prepared and stained with hematoxylin and eosin (H&E) and Masson’s trichrome stains. The histologic sections were examined using light microscopy at x100 and x400 magnifications. Numbers of osteoblasts and blood vessels were counted in sections. New mineralized tissue formation and blood vessel area were measured using Olympus Soft Imaging System AnalySIS FIVE^®^ digital imaging software (Olympus Optical Co. LTD, Tokyo, Japan). Five fields were randomly selected in each section and the calculated area was expressed as mean % ± SD % of the corresponding field of view.

### Biochemical analyses

Following 12 hours of fasting, intracardiac blood samples were collected into EDTA vacutainer tubes. Samples were centrifuged at 3000 g for 10 min at 4°C and plasma was stored in aliquots at -80°C. The AOPP was measured by spectrophotometry on a microplate reader (UV-1601 Visible Spectrophotometer, Shimadzu Corp., Kyoto, Japan). The absorbance was read at 340 nm. Concentrations of AOPP were expressed as μmol/L chloramine-T equivalents, which were abbreviated to μmol/L. For MDA assay, the supernatant absorbance was measured at 535 nm. MDA values were expressed as nmol/mL using a molar extinction coefficient of 1.56x105 M^-1^ cm^-1^ ^2^. Superoxide dismutase activity was evaluated by using BMassay Rat Super Oxide Dismutase ELISA Kit (Bio-Medical Assay Co., Ltd, Beijing, China). The absorbance was measured with an ELISA reader at 450 nm (Rayto Spectrophotometer RT-1904C, Rayto Life and Analytical Sci, Co, Ltd, Shenzhen, China) and a standard curve was used.

### Statistical analysis

Statistical Package for Social Sciences (SPSS) for Windows, Version 15.0 (SPSS Inc, Chicago, USA) software was used in this study. Since all of the variables consisted of numerical values, data were first evaluated with descriptive statistical methods such as mean, median, variance and standard deviation. Kolmogorov–Smirnov test was used to check whether distribution of the sample is consistent with normality assumptions. Levene’s test was employed to assess the homogeneity of variances. For variables that meet the criteria for normal distribution, the Analysis of Variance (ANOVA) test was used for multiple comparisons. *Post-hoc* tests; Tukey’s Honestly Significant Difference (HSD) Test or Tamhane T2 were performed for pairwise comparisons for equal and unequal variances, respectively. Kruskal-Wallis and Mann-Whitney U test with Bonferroni correction were applied for variables that did not meet the criteria for normal distribution. The comparisons between two independent groups were performed using the Independent-Samples T test or Mann-Whitney U test depending on the distribution of the data. For the assessment of dependent variables that were normally distributed, Paired-Samples T test was used, which was substituted by Wilcoxon Signed Rank Test when dependent variables are not normally distributed. The results were evaluated in a confidence interval of 95% and p<0.05 was considered statistically significant. The *p* value was adjusted to p^1^<0.003 when Mann-Whitney U test with Bonferroni correction was applied.

## RESULTS

### Body weight and fasting blood glucose measurements

Mean body weights of all diabetic rats (239.74±23.53 g and 254.76±28.16 g at 10^th^ and 30^th^ days, respectively) significantly decreased when compared with their initial mean body weights (295.3±11.9 g and 292.07±11.28 g at 10^th^ and 30^th^ days, respectively) (p<0.05). On the other hand, a significant weight gain pattern was detected in non-diabetic groups (300.38±11.04 g, 329.07±11.36 g, at 10^th^ and 30^th^ days, respectively) compared with their initial mean body weights (291.42±8.37 g and 287.35±3.38 g at 10^th^ and 30^th^ days, respectively) (p<0.05). The mean value of fasting blood glucose level in diabetic groups (366.91±63.56 mg/dl) was significantly higher than the baseline values (85.65±8.18 mg/dl) (p<0.05).

### Biochemical evaluation

A significant difference was found between the study groups at the 10^th^ day in AOPP levels. The mean of DM group was significantly higher than those of ETN, MEL, and CONT groups (p<0.05). At the 30^th^ day, the mean of DM group was found to be significantly higher than the DM+MEL, ETN, MEL, and CONT groups (p<0.05). Furthermore, the mean of DM+ETN group was higher than that of DM+MEL group (p<0.05) (Table 1). At the 10^th^ day, MDA levels were observed to be significantly higher in DM group than those of ETN, MEL, and CONT groups (p<0.05). At the same time, DM+ETN group demonstrated significantly higher levels of MDA than ETN, MEL, and CONT groups (p<0.05). At the 30^th^ day, significantly higher level of MDA was detected in DM group when compared with DM+MEL, ETN, MEL, and CONT groups (p<0.05) (Table 1). The median of SOD in DM group was significantly higher than that of DM+ETN group at the 10^th^ day (p^1^<0.003). At 30^th^ day, the mean SOD levels in DM, DM+MEL, and DM+ETN groups were lower than those of ETN, MEL, and CONT groups (p<0.05) (Table 1).

### Light microscopy observations

#### Osteoblast count

**Figure 2:**
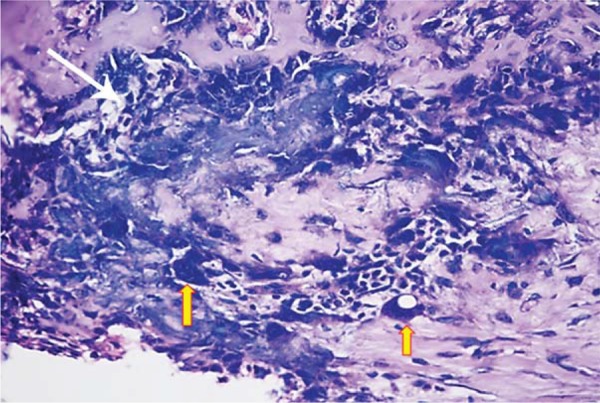
Irregularly arranged osteoblasts (thin white arrow) and large multinucleated osteoclast-like cells (thick yellow arrows) observed in the section of DM group at the 10th day (hematoxylin & eosin x400)

**Figure 6:**
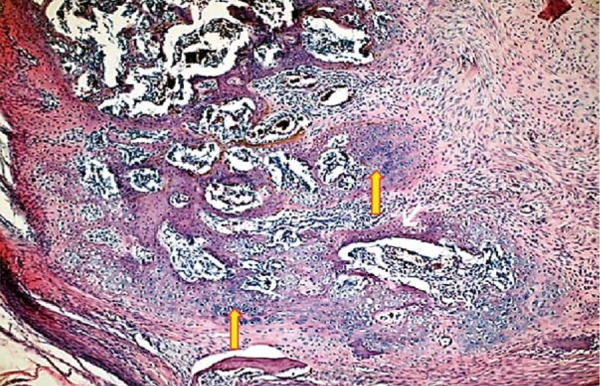
At the 30th day, endochondral bone formation (thick yellow arrows), osteoblasts (thin white arrow), and fibrosis area were detected around the defect in DM+MEL group (hematoxylin & eosin x100)

**Figure 7:**
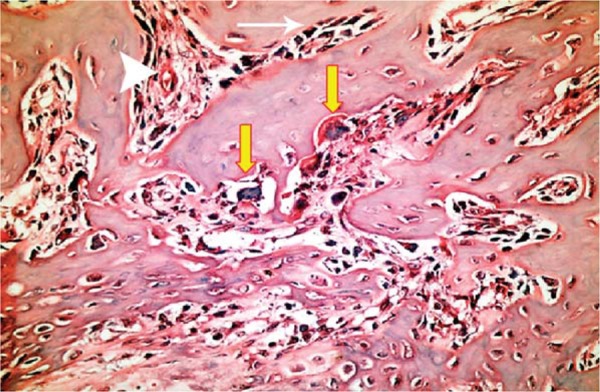
At the 10th day, osteoblasts (thin white arrow), osteoclast-like cells (thick yellow arrows), and blood vessels (triangular white arrowhead) were observed around new mineralized tissue in the defect area of the section obtained from CONT group (hematoxylin & eosin x400)

At the 10^th^ day, a significant decrease in the mean number of osteoblasts was detected in DM group when compared with those of DM+MEL, ETN, MEL, and CONT groups (p<0.05). The mean osteoblast count in DM+MEL group was found to be significantly higher than that of DM+ETN group, whereas it was significantly lower than the mean number of osteoblasts in ETN, MEL, and CONT groups (p<0.05). In non-diabetic groups, young osteoblasts, cuboidal and robust, were observed in a regular arrangement at the 10^th^ day (Figure 7). However, in diabetic groups, uneven shaped osteoblasts were irregularly lined up and were found to be disorganized (Figure. 2). It was noted that the new mineralized tissue formation had been almost completed at the 30^th^ day (Figure 6).

## Number and area of blood vessels

In 10^th^ day specimens, the mean number of blood vessels and mean area occupied in sections were both significantly lower in DM group than those measured in DM+MEL, ETN, MEL, and CONT groups (p<0.05). Although DM+MEL group demonstrated a significant increase compared with DM+ETN group in both variables (p<0.05), these were still lower than those of non-diabetic groups (p<0.05). In 30-day-old specimens, the mean number and area of blood vessels were significantly higher in non-diabetic groups than those calculated in diabetic rats (p<0.05, Table 2).

**Table 2:** Measurement of osteoblast, new mineralized tissue area, and blood vessel in study groups. Data were expressed as mean±standard deviation. (DM: Diabetes Mellitus; MEL: Melatonin; ETN: Ethanol; CONT: Control)

Day	n	Groups	Osteoblast Count	New Mineralized Tissue Area (mm^2^)	Blood Vessel Count	Blood Vessel Area (mm^2^)
10	8	DM	157.5±22.26^A,B,C,D^	0.16±0.08^A,B,C,D^	18±5.98^A,B,C,D^	0.0043±0.0007^A,B,C,D^
10	8	DM+MEL	227.5±33.9^A,E,F,G,H^	0.39±0.11^A,E,F^	30.75±6.36^A,E,F,G,H^	0.0125±0.001^A,E,F,G^
10	7	DM+ETN	165.42±24.77^E,I,K,L^	0.26±0.05^G,H^	18.28±7.58^E,I,J,K^	0.0034±0.0008^E,H,I,K^
10	7	ETN	291.57±6.45^B,F,I^	0.53±0.14^B,I,K^	42.71±7.3^B,F,I^	0.0171±0.001^B,H,L,M^
10	7	MEL	289.43±8.18^C,G,K^	0.64±0.12^C,E,G,I^	46.28±6.23^C,G,J^	0.0174±0.0018^C,F,I,L^
10	7	CONT	289.42±10.64^D,H,L^	0.63±0.14^D,F,H,K^	46.57±6.7^D,H,K^	0.0176±0.0022^D,G,K,M^
30	7	DM	127.57±14.66	0.45±0.06(0.43)^A,B,C^	9.42±2.99^A,B,C^	0.0022±0.0005^A,B,C^
30	7	DM+MEL	116±25.94	0.52±0.09(0.56)^D,E^	9.57±4.11^D,E,F^	0.0031±0.0023^D,E,F^
30	7	DM+ETN	127.71±22.02	0.47±0.11(0.44)^F,G^	10.14±1.77^G,H,I^	0.0024±0.0005^G,H,I^
30	7	ETN	150.57±10.98	0.79±0.11(0.78)^A^	24.85±2.11^A,D,G,K^	0.0095±0.0018^A,D,G^
30	7	MEL	152.28±21.14	0.82±0.03(0.81)^B,D,F^	33.14±12.38^B,D,E,H^	0.014±0.0027^B,E,H^
30	7	CONT	151.28±46.97	0.81±0.04(0.8)^C,E,G^	32.28±7.25^C,E,F,I,K^	0.0142±0.0028^C,F,I^

Upper-case letters in the same column indicate significant difference between two groups at the same time points

## New mineralized tissue formation

**Figure 3:**
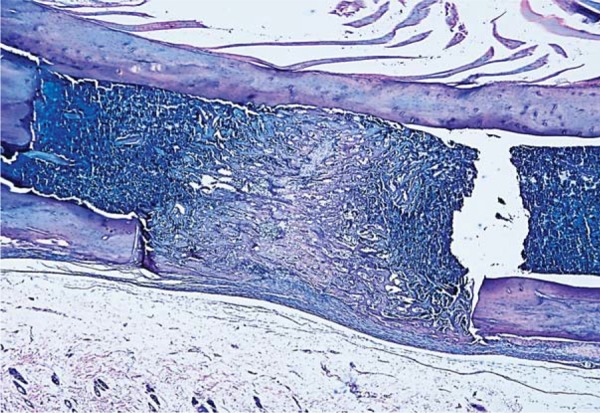
Histological view of the defect that is nearly filled with new mineralized tissue in DM group at the 30th day (hematoxylin & eosin x40)

**Figure 4:**
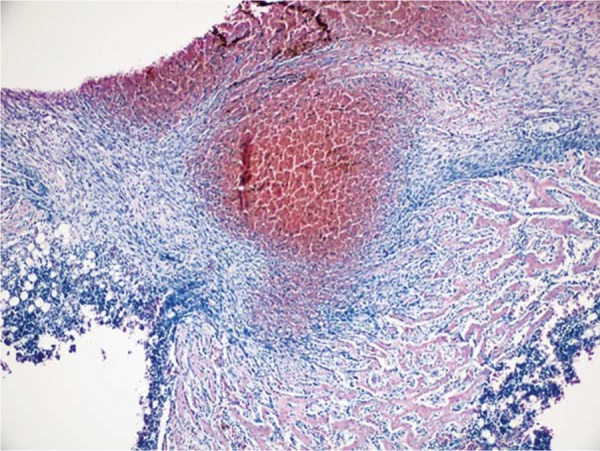
At the 10th day, hematoma formation was apparent in the defect area surrounded by fibrosis and thin trabeculae of new mineralized tissue in DM+MEL group (hematoxylin & eosin x100)

**Figure 5:**
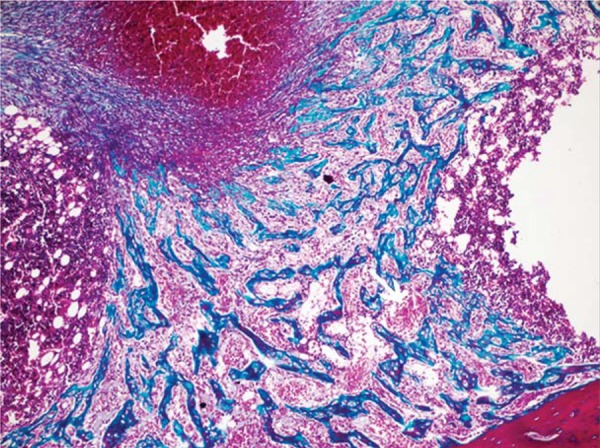
Fibrosis and mineralized tissue formation in the defects of DM+MEL groups at the 10th day (Masson’s trichrome x100) (Arrow: Blood Vessels)

In 10^th^ day specimens, the mean area of new mineralized tissue formation was significantly lower in DM when compared with DM+MEL, ETN, MEL, and CONT groups (p<0.05). Furthermore, the means of DM+MEL, DM+ETN, and ETN groups were significantly lower than those of MEL and CONT groups (p<0.05). In 30^th^ day specimens, the mean area of new mineralized tissue was significantly lower in DM group than in ETN, MEL, and CONT groups specimens (p^1^<0.003) (Figure 3). In addition, the means of DM+MEL and DM+ETN were significantly lower than those of MEL and CONT groups (p^1^<0.003). In 10^th^ day specimens, hematoma formation was clearly visible in the center of bone defects. In DM+MEL groups, the hematoma was surrounded by fibrosis area and thin trabeculae of new mineralized tissue (Figure 4 and Figure 5).

## DISCUSSION

Diabetes mellitus poses a significant threat to the quality of life and constitutes a considerable health-care burden. On 2014, approximately 387 million people worldwide were living with DM, which will probably reach 592 million by the year of 2035^16^. Therefore, experimental research on DM is a valuable adjunct to scientific arsenal to elucidate different aspects of this disease. Various animal species have been used for this purpose, but male rodents, especially rats, are generally preferred because of their hormonal characteristics, high reproduction rates, availability at reasonable cost and ease of maintenance. Commercial availability, the presence of well-established methodology and low animal mortality rates have made the STZ the agent most commonly employed to induce DM. Accordingly, we administered a single dose of 65 mg/kg i.p. STZ to induce irreversible DM in male rats. Cellular proliferation has been reported to reach its peak during the first week post-injury, followed by the maturation of cellular components and endochondral ossification, which occurs near the end of second week. In third week post-injury, the osteoblastic activity has been shown to gradually decrease and woven bone is replaced by lamellar bone^13^. In experimental Type 1 DM models, waiting period of four weeks after the induction of DM is a common protocol to establish diabetic condition and to let the harmful effects of DM on various organs to appear. After eight weeks, these effects have been reported to become more pronounced^1^. Accordingly, we selected the sacrifice time points as 10^th^ and 30^th^ post-operative days to exploit the advantage of concurrently using both well-established models of bone defect healing and experimental Type 1 DM. To avoid spontaneous tibia fractures that may lead to subsequent infection and create an easily reproducible bone defect, we used a commercially available 2.1 mm round dental bur. Diabetes mellitus has been associated with oxidative stress, which leads to impaired wound healing process due to decreased inflammatory response, lack of well-organized granulation tissue, decreased amount of growth factors, poor angiogenesis, and altered collagen organization. Structural and biochemical damages in cellular components that participate in the healing process in DM necessitate multi-disciplinary research on substances that may overcome the deleterious effects of this disease on osseous healing, either by reducing oxidative stress or by promoting bone healing process. Melatonin hormone may be considered as a viable option because of its free-radical scavenging properties. Melatonin content in biological fluids gradually increases in the early part of the night, reaches its peak level at midnight and decreases just before dawn. Claustrat, et al.^12^ (2005) reported that melatonin secretion is related to the duration of darkness, reaching a peak at 03:00 to 04:00 h. Timing of melatonin administration was selected as 19:00 hours to be consistent with previous studies that have been conducted in rodents^26^ ^,^ ^27^. Melatonin dose was calculated according to the solubility of melatonin powder in ethanol and individual body weight of each animal^26^. In order to determine the extent of oxidative stress and to mediate damages and the effectiveness of antioxidant defense mechanisms that may affect bone healing, we analyzed plasma levels of AOPP, MDA, and SOD, which are biomarkers previously studied^20^ ^,^ ^30^.

Protein and lipid oxidations in clinical or experimentally-induced DM may cause inactivation of antioxidant defense enzymes, thereby leading to structural and functional damages to plasma proteins and lipids. In our study, DM induction alone caused some significant alterations in oxidative stress biomarkers. We have found that plasma levels of AOPP were higher in diabetic groups at both time points. Consistently, plasma level of AOPP has been shown to increase in DM in various study designs^2^ ^,^ ^10^ ^,^ ^23^. Çakatay^10^ (2005) reported that subjects with poorly controlled glycaemia had higher plasma levels of AOPP than those having well-regulated DM. Diabetes mellitus is considered to be a major cause of autoxidative glycosylation, free radical formation as well as protein and lipid oxidation. This process increases intracellular formation of glycated biomolecules and specific end products such as AOPP. On the other hand, MDA levels in DM have been associated with the duration of hyperglycemia^2^ ^,^ ^20^ ^,^ ^30^. We detected a significant increase in MDA levels in the plasma samples obtained from DM groups in our study. Membrane lipids are of utmost importance for the preservation of cell integrity. Lipid peroxidation may lead to enzyme inactivation, cross-link of membrane lipids and proteins as well as cell death. In DM, the accumulation of Nicotinamide Adenine Dinucleotide Phosphate (NADPH), one of the glucose metabolites formed as a result of glucose oxidation, enhances lipid peroxidation via cytochrome P-450 system. Malondialdehyde, which is the end product of lipid peroxidation, has been found to increase in DM^11^. The activity of SOD in DM group at the 30^th^ day was significantly lower than MEL and CONT groups. Our result was consistent with previous studies that reported that plasma SOD enzyme activity was lower in diabetic groups than the controls^28^. The decrease of SOD activity in diabetic groups may be attributed to the increase of glycosylated SOD, which can lead to the inactivation of this enzyme at the end of eight weeks^2^.

Melatonin administration caused a significant decrease in AOPP plasma and MDA levels in diabetic rats at the 30^th^ day. This finding could be explained by the time dependent influence pattern of melatonin to exhibit its antioxidant properties on lipid metabolism^22^. Reactive oxygen species are by-products of partial O_2_ reduction during ATP synthesis. Hydroxyl radical (HO), which is formed during this process, is one of the most aggressive radicals that reacts with proteins and lipids. As enzyme systems could not efficiently detoxify HO, antioxidants, which directly scavenge this radical, are required to support the antioxidative defense system. Melatonin, as a receptor-independent free radical scavenger and a broad-spectrum antioxidant, was found to be a potent HO scavenger. The interaction of melatonin with free radicals generates metabolites that contribute to the reduction of lipid and protein oxidation. Therefore, melatonin doses administered in our study may have led to the decrease in the plasma levels of AOPP and MDA. On the other hand, there are conflicting arguments concerning the changes in SOD levels measured in the presence of DM and melatonin supplementation. No consensus could be reached on this particular subject, since some studies indicate higher levels of SOD^20^ whereas others report reduction in the enzyme activity^3^. Considerable inconsistencies that exist between the sample types and experimental designs used in these studies probably contribute to the discrepancies reported in their findings. In our case, the mean plasma level of SOD in DM+MEL group was lower than that of the DM group at the 10^th^ day; however, this difference did not reach the level of statistical significance. Besides other factors, our limited sample size and strict statistical methodology may have also influenced the study outcome.

The histological characteristics and healing patterns of hard tissue wounds in DM have been investigated using surgical bone defects or fractures created in different anatomic regions of small animal models. Azad, et al.^7^ (2009) reported that diabetic rats having a segmental femoral defect had a significantly lower area of mineralized tissue when compared with non-diabetics at three-week period. Hamann, et al.^17^ (2013), who evaluated the bone formation in femoral bone defects of diabetic and non-diabetic rats at twelve weeks, have found that diabetic animals had significantly lower amount of new mineralized tissue when compared to their non-diabetic counterparts. In a study conducted by Shyng, et al.^29^ (2001), a small number of flattened and inactive osteoblasts were detected overlying the immature bone surfaces in the histological observations of calvarial defects at three-week period. In addition, they demonstrated that necrotic bone had persisted at the defect margins, which indicates a deformity in the remodeling phase. Their findings suggest that the presence of DM may disrupt mineralization, resorption or remodeling phases during bone healing. Follak, et al.^15^ (2004) observed a reduced and retarded mineralization process in the first two weeks of bone healing in poorly compensated diabetic animals. Similarly, Picke, et al.^22^ (2015) reported lower bone formation rate in diabetic rats when compared with controls and suggested that DM may have caused histologic defects in osteoblasts. Fracture healing may also be compromised in DM. Beam, et al.^9^ (2002), who evaluated the effects of DM on the early and late phases of fracture healing in diabetic rats, have found that cell proliferation in the hard callus of diabetic rats decreased to a greater extent than that of controls at the 10^th^ day. Also, the cartilage area, as well as the size and number of proliferating chondrocytes, were found to be significantly lower in diabetic rats. The authors reported an obvious delay in the endochondral ossification of fractures in diabetic rats at eight-week time point. Kayal, et al.^19^ (2009) observed smaller cartilage area and more prominent osteoclastogenesis in diabetic mice with artificial femoral fractures than those of controls at the 10^th^ day. The amount of new mineralized tissue formation in diabetic mice at 16^th^ and 22^th^ days were observed to be significantly less than those of controls, suggesting that diabetes causes a reduction in the endochondral bone formation during fracture repair. Our findings at the 10^th^ day have shown that the new mineralized tissue formation, number of osteoblasts and new blood vessels, as well as the area occupied in sections, were significantly lower in DM groups without melatonin supplementation when compared with controls. Although it is not possible to directly compare these results with those of aforementioned studies because of differences in the design concepts and the animal species used, we think that our findings represent a similar pattern, which indicates deleterious effects of DM on the healing of bone defects, particularly in the early phase of the process.

Melatonin is thought to affect bone metabolism by promoting the proliferation of osteoblasts and the synthesis of osteoprotegerin, which leads to the inhibition of the differentiation of osteoclast-like cells. When investigated in non-diabetic animals, Satomura, et al.^27^ (2007), who administered i.p. melatonin to mice, have found a significant increase in the ratio of new to old bone mass in the surface of femoral cortex. In similar experimental settings, Koyama, et al.^21^ (2002) reported higher bone mass and trabecular thickness, but lower osteoclast surface and number in growing young mice treated with melatonin for four weeks. Authors concluded that the melatonin administered in pharmacologic doses had increased bone mass predominantly through suppression of bone resorption. Experimental designs that include supplemental melatonin administration in the presence of bone defects created in diabetic subjects are very limited. Yousuf, et al.^31^ (2013) locally applied melatonin to artificial periodontal defects created in the mandible of STZ induced diabetic rabbits. They reported significantly higher osteoblast count at three and six weeks, and, at the same time points, lower number of osteoclasts in melatonin treated DM groups when compared with non-treated diabetic rabbits. Authors attributed the increase in the amount of new mineralized tissue they have found to the aforementioned changes in the cell count balance that shifts towards osteoblast formation. Kaya, et al.^18^ (2013) evaluated the effects of melatonin administration on bone defect healing process in diabetic rats. They demonstrated that melatonin-treated diabetic rats had significantly increased values of osteogenesis indicators, but decreased number of inflammation markers when compared with those of non-diabetic rats. Consistent with these studies, our findings at the 10^th^ day showed that DM+MEL group had higher number of osteoblasts, as well as new mineralized tissue formation area, but lower osteoclast-like cell count when compared with DM group. The evaluation of the number and area of blood vessels showed that DM group had significantly decreased values compared with those of non-diabetic groups at 10^th^ and 30^th^ days. These findings support the fact that DM may have impaired new vessel formation. Similarly, Altavilla, et al.^4^ (2001) have shown that the level of lipid peroxidation is inversely correlated with new vessel formation in diabetic mice. On the other hand, DM+MEL group had significantly higher number and area of blood vessels at the 10^th^ day when compared with DM group, which is in accordance with studies^29^ that present significantly higher number of blood vessels in melatonin-administered rats than those of control group in every phase of wound healing. These findings could suggest that melatonin administration is capable of ameliorating the deleterious effects of DM on blood supply.

## CONCLUSION

Within the limits of this experimental study, the administration of supplemental melatonin in diabetic rats with hard tissue defects has demonstrated positive histologic effects on the early stage of bone healing and exhibited limited free-radical scavenging properties, as evidenced by the changes in the plasma levels of oxidative stress-related biomarkers.
